# Case Report: Isolated lingual pain: a rare and atypical presentation of trigeminal neuralgia successfully treated with microvascular decompression

**DOI:** 10.3389/fpain.2026.1780537

**Published:** 2026-05-07

**Authors:** Yang Xu, Yuqian Li, Yansong Zhang, Yongyan Chen, Zhiyi He, Shuaishuai Zhu, Zhengxiang Luo

**Affiliations:** 1 Department of Neurosurgery, the Affiliated Brain Hospital of Nanjing Medical University, Nanjing Medical University, Nanjing, China; 2 Department of Neurology, the Affiliated BenQ Hospital of Nanjing Medical University, Nanjing, China

**Keywords:** glossopharyngeal neuralgia, microvascular decompression, misdiagnose, neurosurgery, trigeminal neuralgia

## Abstract

**Background:**

Trigeminal neuralgia (TN) typically presents with paroxysmal, shock-like, or stabbing pain in the unilateral facial region corresponding to the trigeminal nerve distribution. However, cases manifesting solely as isolated tongue pain are exceedingly rare.

**Case presentation:**

A 63-year-old female presented with a two-year history of left-sided tongue pain. She was initially suspected of having glossopharyngeal neuralgia (GPN) and treated with oxcarbazepine at the local hospital, which provided suboptimal relief. Over the past two months, her pain intensified significantly. Upon referral to our department, a comprehensive evaluation was conducted—including a detailed history, neurological examination and high-resolution magnetic resonance imaging (MRI). However, we ruled out the diagnosis of glossopharyngeal neuralgia and strongly believe the patient suffered from trigeminal neuralgia. First, MRI imaging revealed vascular compression of the trigeminal nerve. Then, glossopharyngeal nerve block helped rule out glossopharyngeal neuralgia. Most importantly, intraoperative exploration confirmed definite vascular compression of the trigeminal nerve, while vessels were observed adjacent to the glossopharyngeal nerve without significant compression. The patient subsequently underwent left microvascular decompression (MVD) of the trigeminal nerve. Postoperatively, her pain resolved completely.

**Discussion and conclusion:**

Trigeminal neuralgia and glossopharyngeal neuralgia share overlapping clinical features, pathogenesis, imaging findings and triggering factors. These two diseases are typically distinguished by their characteristic pain locations. This case illustrates a diagnostically challenging presentation of TN, with pain confined exclusively to the tongue, which is highly prone to being misdiagnosed as glossopharyngeal neuralgia. The definitive identification of neurovascular compression on imaging, coupled with the complete resolution of pain following MVD, conclusively confirmed the diagnosis of TN. This report emphasizes that clinicians should meticulously analyze pain distribution and correlate it with imaging evidence of vascular compression to accurately differentiate between these two entities, thereby avoiding misdiagnosis and mitigating the risk of unnecessary interventions or secondary injury.

## 1 Introduction

Trigeminal neuralgia (TN) is a common cranial nerve disease characterized by paroxysmal, severe, shock-like or stabbing pain confined to the distribution of one or more branches of the trigeminal nerve (1). According to the International Classification of Headache Disorders (ICHD-3), classical primary TN is most frequently attributed to pulsatile vascular compression at the root entry zone (REZ) of the trigeminal nerve. This established pathophysiological basis has cemented microvascular decompression (MVD) as its most effective curative treatment (2, 3).

The causes of TN can be classified into three types: classic type, secondary type and idiopathic type. The classic type is caused by the compression of intracranial vessels at the REZ area of the trigeminal nerve. The secondary type of TN is often accompanied by other neurological symptoms and is usually associated with multiple sclerosis and pontocerebellar region tumors. The idiopathic type of TN indicates that no clear neurological cause can be found (4).

Clinically, the pain of the majority of patients with trigeminal neuralgia is merely located on the face, most commonly affecting the second branch (maxillary branch) and the third branch (mandibular branch). The typical “trigger point” and the pain patterns triggered by daily activities (such as chewing, washing the face) usually make the diagnosis relatively clear (5). However, when the pain presents as an atypical location or feature, the diagnosis will face challenges. Pain that is predominantly or completely limited to the lingual nerve distribution (the anterior two-thirds of the tongue) represents a highly atypical clinical picture. Such isolated lingual pain is exceedingly rare in the published papers. Wu (6) once reported a case misdiagnosed as glossopharyngeal neuralgia: the patient had tongue pain, which persisted after glossopharyngeal nerve microvascular decompression but resolved following trigeminal nerve decompression after a correct diagnosis of trigeminal neuralgia.

This atypical distribution of pain is highly prone to misdiagnosis. In the process of differential diagnosis, glossopharyngeal neuralgia (GPN) is usually the first consideration, as it typically presents with pain in the base of the tongue, the pharynx, and the deep part of the ear (7, 8). Although both diseases share high similarities in their pathogenesis (vascular compression), imaging manifestations (neurovascular conflict), and treatment methods (MVD), incorrect diagnosis can still lead to a failure in selecting the surgical target, preventing patients from achieving the best therapeutic effect.

Therefore, this report aims to present a case of primary trigeminal neuralgia that was successfully treated with microvascular decompression and manifested exclusively as isolated lingual pain. By detailing the clinical presentation, diagnostic reasoning, key imaging findings, and confirmatory surgical outcome, we seek to explore the unique anatomical basis of this presentation. Furthermore, we emphasize the critical importance of including trigeminal neuralgia in the differential diagnosis when evaluating unexplained unilateral tongue pain. with the goal of enhancing clinicians’ recognition of this rare manifestation and preventing misdiagnosis or missed diagnosis. We hope that our case report will help clinicians enhance their understanding of such rare manifestations and avoid misdiagnosis and missed diagnoses.

## 2 Case description

### 2.1 History

A 63-year-old woman was admitted due to intermittent severe pain in the left side of her tongue. The pain was triggerred by chewing and swallowing, with each episode lasting from several minutes to tens of minutes. Treatment with oxcarbazepine had provided partial relief initially, however, the pain worsened over the past two months and dose escalation failed to provide adequate control. She had been initially diagnosed with glossopharyngeal neuralgia at a local hospital based on her symptomatology. The patient expressed great anxiety and distress due to the unclear diagnosis and persistent pain, and was eager to obtain effective treatment to relieve the pain and return to normal life. He also mentioned that he initially suspected his pain was related to dental problems, but the symptoms did not improve after dental examination and treatment.

### 2.2 Clinical examination

After detailed questioning, the pain was predominantly localized to the junction between the middle and posterior regions of the left tongue. There was no abnormal sensation in the face. Also, the corneal reflex was present. The chewing muscle strength was normal and no obvious “trigger points” were felt on the face. Local lesions of the tongue (such as ulcers, tumors) and dental-related diseases were excluded.

### 2.3 Imaging findings

**Figure 1:**
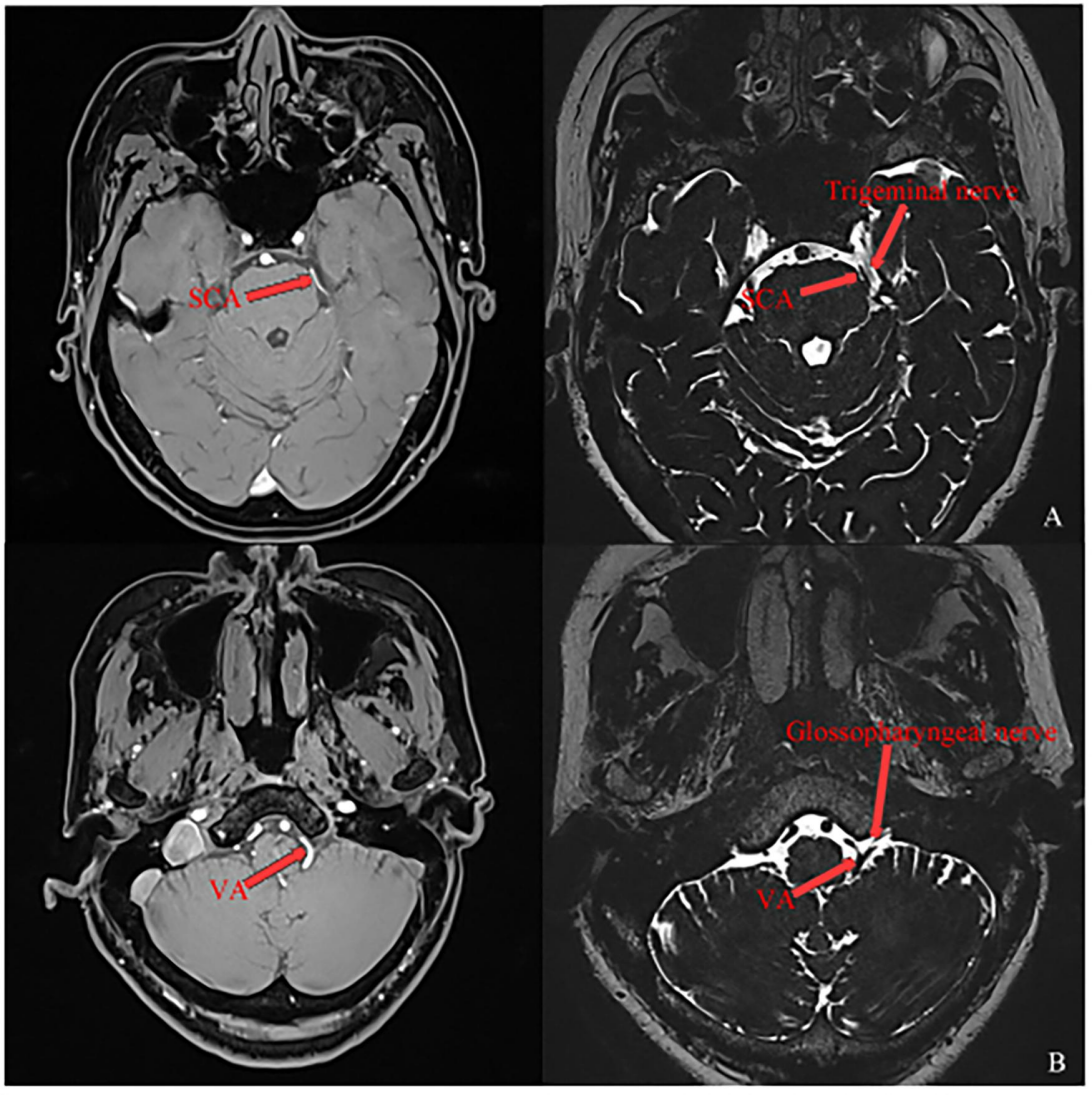
**(A)** the left trigeminal nerve has a close relationship with the surrounding blood vessels. **(B)** The left glossopharyngeal nerve has a close relationship with the surrounding blood vessels.

Magnetic resonance imaging (MRI) revealed a vascular loop (presumed to be the superior cerebellar artery) in close contact with the left trigeminal nerve at its root entry zone (REZ), particularly compressing its inferolateral aspect (corresponding to the mandibular division fibers) (Figure 1A). Interestingly, there are also blood vessels accompanying the glossopharyngeal nerve(presumed to be the vertebral artery) (Figure 1B).

### 2.4 Differential diagnosis

Glossopharyngeal neuralgia was considered. In the sensory innervation of the tongue, the anterior two-thirds are supplied by the mandibular division of the trigeminal nerve, while the posterior one-third is innervated by the glossopharyngeal nerve. The patient's pain localized to the central part of the tongue lies in the transitional zone between the territories of the trigeminal and glossopharyngeal nerves. Anatomically, this region falls within the trigeminal nerve distribution. Other conditions, such as lingual tumors and dental pulp diseases, were also ruled out.

### 2.5 Diagnostic block

To further clarify the diagnosis, we also conducted a glossopharyngeal nerve block experiment. We prepared 2 mL of 2% lidocaine and sprayed it onto the painful area of the tongue. We instructed the patient to keep the mouth open and tilted backward. After 5 min, the patient spat out the liquid in the throat. The patient reported numbness in the oropharynx, but the pain in the tongue remained. Based on these findings, the diagnosis of glossopharyngeal neuralgia could be effectively ruled out.

### 2.6 Treatment and surgical findings

When pharmacological therapy proves ineffective, microvascular decompression (MVD) is currently the optimal surgical intervention for cranial neuralgias caused by intracranial neurovascular compression. Studies indicate success rates of 90.3% for TN and 86.7% for GPN treated with MVD (9, 10). If the procedure fails, it is imperative to consider the possibility of missed areas of compression during exploration or an incorrect initial diagnosis.

Preoperative cranial MRI in this patient revealed close relationships between vascular structures and both the left trigeminal and glossopharyngeal nerves. During surgery, thorough exploration was performed. The responsible vessel compressing the trigeminal nerve, identified as the superior cerebellar artery (SCA), was adequately dissected and decompressed. Postoperatively, the patient's lingual pain resolved completely.

The trigeminal nerve is a large sensory root that emerges from the mid-pons ventrally. Within the cistern, its divisions are topographically organized: the mandibular nerve is located caudolaterally, the ophthalmic nerve rostromedially and the maxillary nerve occupies an intermediate position.

**Figure 2:**
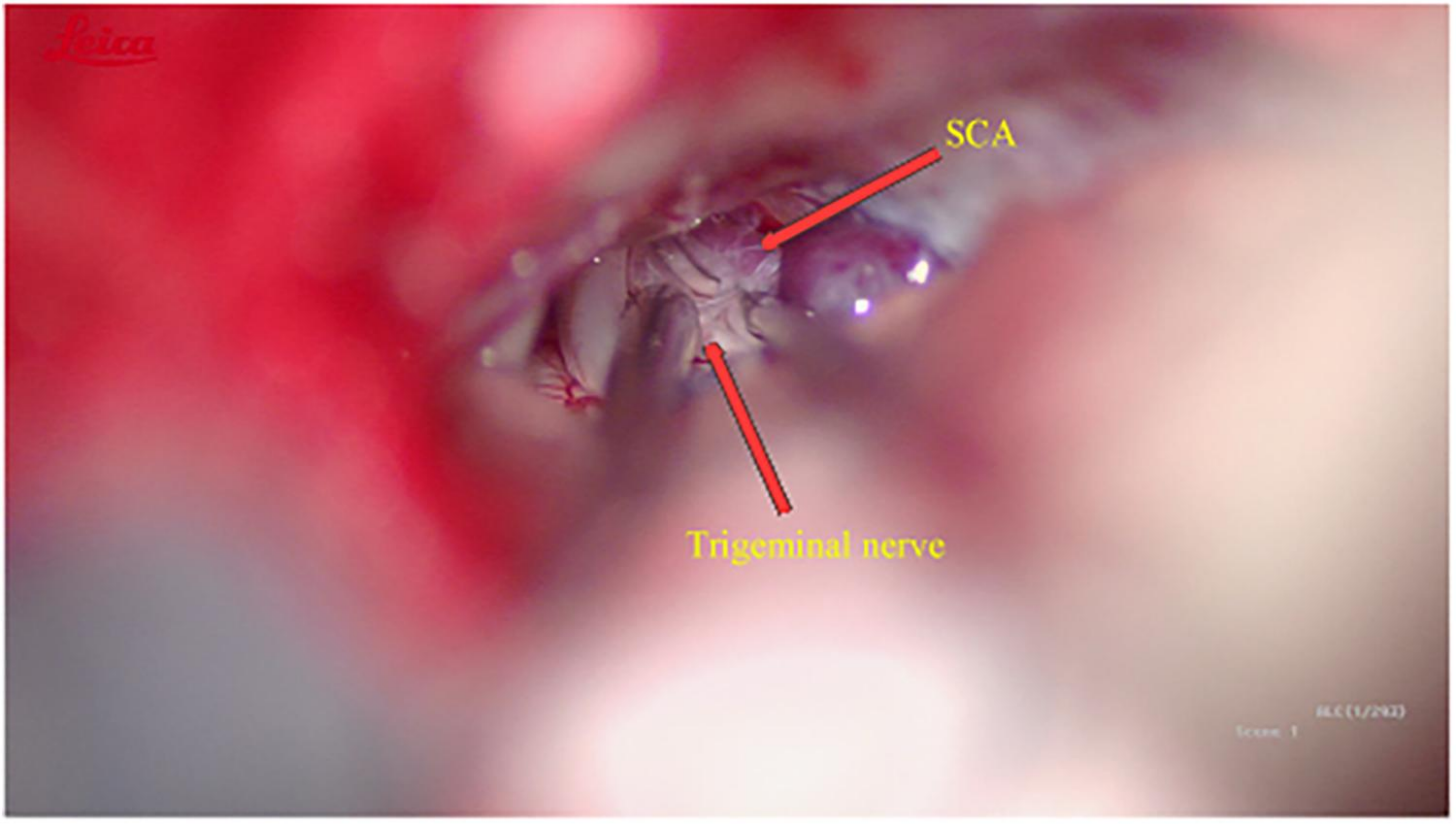
The SCA compresses the trigeminal nerve from its inferolateral aspect.

**Figure 3:**
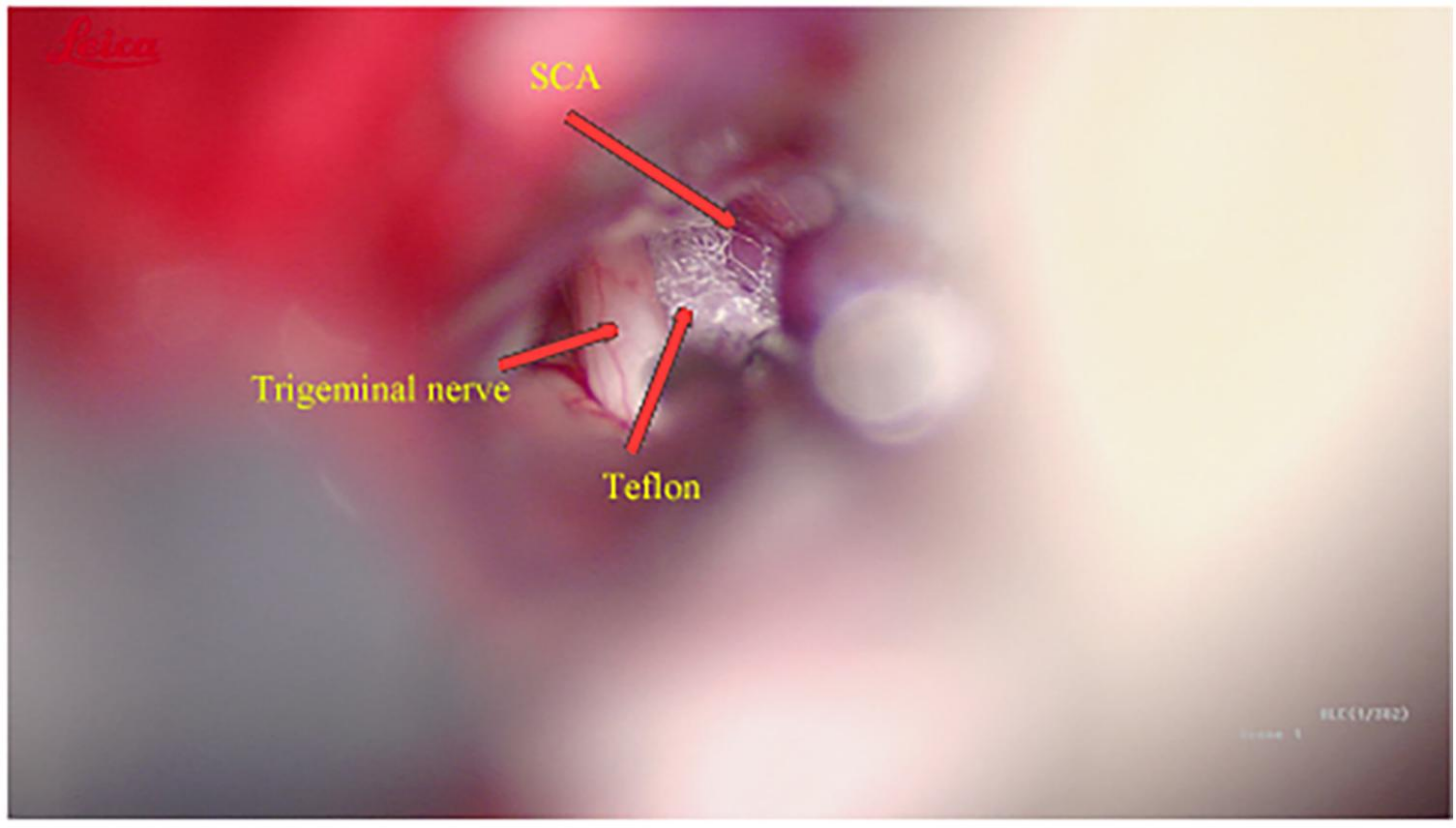
A teflon pledget was interposed to isolate the nerve from the artery.

Intraoperatively, the trigeminal nerve was found to be compressed precisely at its caudolateral aspect by the SCA (Figure 2), providing definitive confirmation of the diagnosis of left-sided trigeminal neuralgia. This anatomical correlation clearly explains the patient's presentation of isolated lingual pain, as this region corresponds to the sensory distribution of the mandibular division. The trigeminal nerve was adequately decompressed using a Teflon pledget (Figure 3).

**Figure 4:**
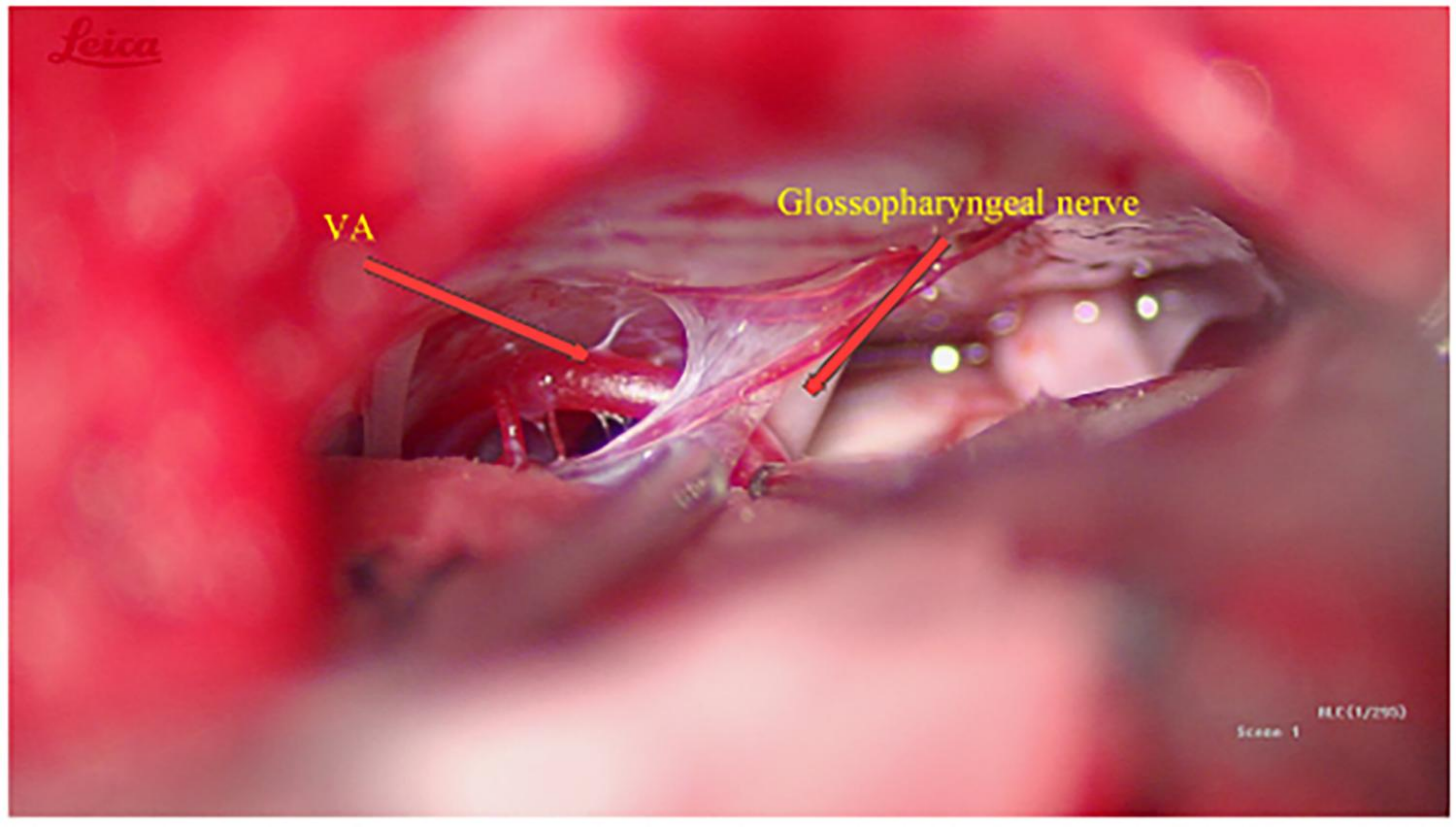
Exploration of the glossopharyngeal nerve.

Interestingly, during the exploration of the glossopharyngeal nerve, a vessel was observed coursing over its surface. However, no significant compression was evident (Figure 4). Consequently, no specific intervention was performed on the glossopharyngeal nerve.

### 2.7 Postoperative course and follow-up

Upon emergence from anesthesia, the patient reported complete resolution of the left-sided lingual pain. No new neurological deficits, such as facial numbness, masticatory weakness, or hearing loss, were observed postoperatively. At the one-month follow-up, the patient remained pain-free without requiring any analgesic medication. During the follow-up, the patient reported that his tongue pain completely disappeared after trigeminal nerve microvascular decompression, and he could eat, speak and sleep normally without any discomfort. He expressed high satisfaction with the treatment effect, stating that the persistent pain which had troubled him for a long time was relieved, and his mental state and quality of life were significantly improved.

## 3 Discussion

The initial diagnostic dilemma in this case is classic. As stated in previous articles, TN and GPN share significant similarities in pain characteristics, etiology of vascular compression and treatment approaches (11, 12). Clinically, the distinction between them primarily relies on the distribution of pain: GPN typically manifests as pain in the base of the tongue, pharynx, tonsillar fossa, or deep ear, often triggered by swallowing. However, classical TN is most often reported in areas including the mid-face, jawline, and due to the mandibular nerve's lingual branch–the anterior two-thirds of the tongue. The pain in this case precisely fell within this overlapping and ambiguous zone—the tongue. General sensation from the anterior two-thirds of the tongue is supplied by the lingual nerve of the mandibular division of the trigeminal nerve, while the posterior one-third is innervated by the glossopharyngeal nerve (13). Consequently, pain localized to the central area of the tongue, without clear facial trigger points, is highly susceptible to being attributed to GPN, as initially experienced by the patient at the external hospital. The definitive diagnostic process in this case compellingly demonstrates that “lingual pain” is not synonymous with GPN, and meticulous localization is the first critical step in avoiding misdiagnosis.

The diagnosis in this case was not based solely on clinical presumption but was built upon a progressive chain of objective evidence. Firstly, high-resolution magnetic resonance imaging (MRI) indicated vascular compression at the trigeminal nerve root entry zone, providing initial justification for surgical intervention. However, it was noteworthy that the glossopharyngeal nerve also showed a close relationship with adjacent vessels, which introduced diagnostic complexity. Secondly, a preoperative lidocaine blockade test helped to partially alleviate suspicion of glossopharyngeal neuralgia. The most decisive evidence, however, came from the intraoperative findings and postoperative outcome. During surgery, the responsible vessel—the superior cerebellar artery—was clearly observed compressing the inferolateral portion of the trigeminal nerve root, which corresponds precisely to the region where sensory fibers of the mandibular division are concentrated. This precise and isolated compression perfectly explains why pain was confined exclusively to the distribution area of the mandibular nerve's terminal branch (the lingual nerve), while other facial regions remained unaffected. However, there is no absolute correspondence between different compression sites and the corresponding regional pain. Based on our experience, this may be because of changes in intracranial pressure after cerebrospinal fluid release and increased neural activity following arachnoidolysis, leading to the displacement of neurovascular structures before decompression. Furthermore, the immediate and complete resolution of pain following microvascular decompression provided Grade A evidence supporting the diagnosis of trigeminal neuralgia, strongly confirming that vascular compression was the sole etiology of the patient's lingual pain. This complete chain of evidence—integrating clinical presentation, imaging findings, intraoperative observations, and therapeutic outcome—forms a logically rigorous clinical argument.

Although previous paper has documented cases of TN predominantly involving the third division, the pain in most such cases often concurrently affects regions such as the mandible, lower lip, or gingiva (11, 14). In contrast, as demonstrated in the present case, pain that is strictly and exclusively confined to the tongue is exceedingly rare in published reports. This ‘pure’ clinical presentation makes it an excellent exemplar of the isolated involvement of the lingual nerve within the mandibular division. Compared to cases with more diffuse symptoms, our case more precisely localizes the etiology to an isolated compression of the mandibular nerve fibers, thereby providing valuable clinical evidence for understanding the functional somatotopy of the trigeminal nerve.

## 4 Conclusion

Given the close proximity of their innervation territories, misdiagnosis between trigeminal and glossopharyngeal neuralgia is highly susceptible to occur. For neuralgias associated with vascular compression, a careful investigation of the patient's history, precise identification of the target nerve, and comprehensive intraoperative exploration with decompression of all potential offending vessels are essential to avoid the risks of surgical failure and recurrence. The present case of trigeminal neuralgia presenting as isolated lingual pain, due to its rare clinical symptoms, constitutes a classic diagnostic “pitfall.” Through meticulous preoperative evaluation, precise surgical intervention, and a profound understanding of trigeminal nerve anatomy and function, we were able to clarify the diagnosis and achieve complete relief of the patient's suffering. We firmly believe that increasing awareness of such atypical presentations will facilitate earlier and more accurate identification of similar cases in the future, thereby preventing unnecessary patient distress and clinical detours caused by misdiagnosis.

## Data Availability

The datasets presented in this article are not readily available because of ethical and privacy restrictions. Requests to access the datasets should be directed to the corresponding authors.
